# Gene Expression Analysis of Different Organs and Identification of AP2 Transcription Factors in Flax (*Linum usitatissimum* L.)

**DOI:** 10.3390/plants12183260

**Published:** 2023-09-13

**Authors:** Fan Qi, Fu Wang, Chunxiao Xiaoyang, Zhenhui Wang, Yujie Lin, Zhanwu Peng, Jun Zhang, Ningning Wang, Jian Zhang

**Affiliations:** 1 Faculty of Agronomy, Jilin Agricultural University, Changchun 130000, China; fan711998@163.com (F.Q.); 13722052064@163.com (F.W.); xycxttkx@163.com (C.X.); wzhjlau@163.com (Z.W.); linyujie97@163.com (Y.L.); zhangjun@jlau.edu.cn (J.Z.); 2 Information Center, Jilin Agricultural University, Changchun 130000, China; pengzhanwu@jlau.edu.cn; 3 Department of Biology, University of British Columbia Okanagan, Kelowna, BC V1V 1V7, Canada

**Keywords:** gene expression, organ, transcription factor, AP2

## Abstract

Flax (*Linum usitatissimum* L.) is an important oilseed crop widely cultivated for its oil and fiber. This study conducted transcriptome analysis to analyze the gene expression profiles of roots, leaves, stamens, pistils, and fruits in the flax cultivar Longya10. A total of 43,471 genes were detected in the RNA-seq data, with 34,497 genes showing differential expression levels between different organs. Gene expression patterns varied across different organs, with differences observed in expression-regulating genes within specific organs. However, 23,448 genes were found to be commonly expressed across all organs. Further analysis revealed organ-specific gene expressions, with 236, 690, 544, 909, and 1212 genes identified in pistils, fruits, leaves, roots, and stamens, respectively. Gene Ontology (GO) enrichment analysis was performed on these organ-specific genes, and significant enrichment was observed in various biological processes, cellular components, and molecular functions, providing new insights for the specific growth patterns of flax organs. Furthermore, we investigated the expression differences of AP2 transcription factors in various tissues and organs of Longya10. We identified 96 AP2 genes that were differentially expressed in different organs and annotated them into various biological pathways. Our results suggest that AP2 transcription factors may play important roles in regulating the growth and development of flax organs including stress response. In summary, our study provides a comprehensive analysis of gene expression patterns in different organs and tissues of flax plant and identifies potential critical regulators of flax organ growth and development. These findings contribute to a better understanding of the molecular mechanisms underlying flax organ development and may have important implications for the genetic improvement of flax crops.

## 1. Introduction

Flax (*Linum usitatissimum* L.), belonging to the Linaceae family, is a versatile crop used for oil and fiber production [1]. Flaxseeds are rich in unsaturated fatty acids (mainly ω-3) and lignans, as well as easily digestible proteins, dietary fibers, and minerals, contributing to their potential to lower the risk of cancer and cardiovascular diseases [2,3]. Consequently, flax is utilized in pharmaceuticals, food products, animal feed, and is a component in paints and varnishes. In the case of fiber flax, its fiber characteristics, including yield, density, tensile strength, flexibility, and biochemical constituents (such as cellulose and lignin content) [4,5], make it an important source for high-quality textiles, pharmaceuticals, and composite materials in industries of automotive and aerospace. As a result, flax fibers hold a significant position in textile and industrial material production [6,7].

Multicellular plant bodies consist of different organs, each with specific structures and functions to meet the plant’s needs in growth, nutrient acquisition, material transport, and reproduction [8]. For example, roots are underground organs of plants, primarily responsible for absorbing water and minerals from the soil, providing support and anchorage, and storing nutrients and water [9]. Leaves contain many chloroplasts, responsible for photosynthesis, and the leaf epidermis has a protective function, reducing water evaporation and light damage [10]. The stamens of plants are generally located outside the pistils and consist of anthers and filaments, while the pistils contain the stigma, style, and ovary. The male and female gametophytes develop and mature in the ovary and anther, respectively [11,12]. After fertilization, the zygote develops into an embryo, and the ovule develops into a seed, while the ovary develops into a fruit [13,14]. Studying different tissue structures can reveal the mechanisms of cell differentiation, tissue formation, and organ development in plant growth and development and provide insights into important physiological processes such as photosynthesis and water and nutrient absorption [8]. The formation of these different types of organs and tissues and their functional differences largely depend on the expression of specific genes [15]. Moreover, different flax organ tissues exhibit varying levels of stress resistance and practical applications, with some genes ensuring the optimal development of tissue and organs. Although studies in other plants have explored the molecular mechanisms of different genes involved in specific growth and development processes of different organ tissues [16,17,18], there is limited research on specific comparisons between different tissues. Therefore, studying genes with specific expression in different organs and tissues is of great significance for understanding plant growth and development’s regulatory mechanisms and biological functions [15].

During the evolutionary process of plants, many transcription factor families have inevitably emerged through biotic and abiotic stress. Transcription factors can enhance the plant’s ability to resist these stresses by regulating the expression of target genes [19]. The AP2 transcription factor family is a widely distributed transcription factor family in plants. Based on the differences in the AP2/ERF domain and other structural features, the AP2 transcription factor family can be further divided into subfamilies such as AP2, ERF, RAV, and Soloist [19]. They play important regulatory roles in plant growth, development, and stress responses [19].

The AP2 transcription factor family is involved in regulating various biological processes. It regulates seed germination and seedling growth and participates in the development of floral organs, root growth and development, and organ differentiation by regulating gene expression and signal transduction [20,21]. In addition, some members of the AP2 transcription factor family regulate plant nutrient metabolism and storage processes [19]. They regulate key processes such as carbon metabolism, nitrogen metabolism, and photosynthesis and participate in the formation of fruits, changes in fruit skin color, seed development, and accumulation of storage proteins [22,23,24], playing important roles in plant growth and development. Furthermore, under stress conditions such as high temperature, salt-alkali, and drought [20,25,26], AP2 transcription factors are induced to regulate plant hormones such as ethylene and abscisic acid [27,28], participating in important biological processes such as plant morphological changes and stress tolerance. The Ethylene Response Factor (ERF) contains a conserved DNA binding domain and directly interacts with GCC in the ethylene response element [29]. Previous studies have shown that ERF1 and ERF3 expression remained constant in NaCl-stressed tobacco cells, while ERF4 is involved in the regulation of gene expression by stress factors and ethylene-mediated stress signal transduction pathways, with the expression being detected in roots and lower in shoots and leaves [30,31,32]. In a previous study, five NsERF (EREBPs) genes were isolated from *Cryptomeria japonica*, and transgenic tobacco analysis showed that all NsERF genes were induced by ethylene in leaves but weakly expressed in roots [33]. It reported the AP2/ERF transcription factor family is involved in regulating gene expression, hormone signal transduction, and cross-talking pathways in different tissues and organs in plants [34,35].

In our study, we analyzed the gene expression profiles of different organs in Longya10 flax and specifically investigated the AP2 transcription factors in the flax genome and their gene expression patterns. To understand the gene expression patterns and regulatory mechanisms underlying the development and growth in flax, we must obtain comprehensive gene expression profiles and identify differentially expressed genes specific to each organ. Moreover, the study of the AP2 transcription factor family involves important aspects such as structure and function, regulatory networks, growth and development, and environmental stress response. Through in-depth research on the AP2 transcription factor family, we can better understand the mechanisms of plant organ growth, development, and stress response and provide an important theoretical basis and technical support for plant genetic improvement and gene engineering applications.

## 2. Results

### 2.1. Gene Expression in Different Tissues and Organs of Flax Variety Longya10

**Figure 1:**
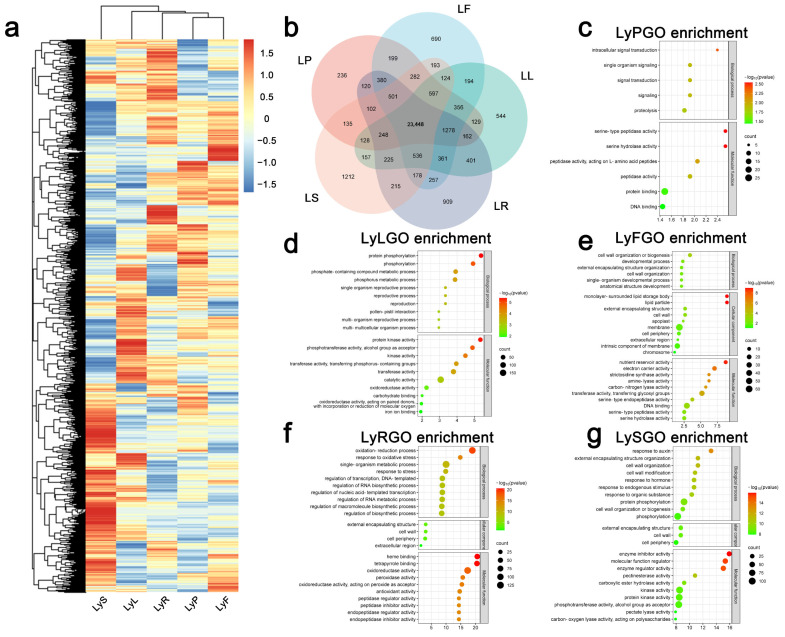
Analysis of whole-genome gene expression profiles in different tissue organs of Longya10 flax. (**a**) Heatmaps were generated to illustrate gene expression levels in different flax tissues. The abbreviations LyR, LyL, LyS, LyP, and LyF represent Longya10 flax roots, leaves, stamens, pistils, and fruits, respectively. Yellow indicates gene expression levels (read counts per million with log2 value). (**b**) Venn diagrams were created to depict common and unique gene expression patterns observed in roots, leaves, stamens, pistils, and fruits of Longya10 flax plants. (**c**) Gene Ontology (GO) classification and enrichment analysis of Longya10 flax pistil-specific expressed genes. (**d**) Longya10 flax leaf-specific expressed gene GO classification and enrichment analysis. (**e**) GO classification and enrichment analysis of Longya10 flax fruit-specific expression genes. (**f**) GO classification and enrichment analysis of Longya10 flax root-specific expressed genes. (**g**) GO classification and enrichment analysis of Longya10 flax stamen-specific expressed genes.

To analyze the gene expression patterns in different organs of flax, we conducted transcriptome analysis to investigate the gene expression profiles of flax organs, including roots, leaves, stamens, pistils, and fruits in the genotype of Longya10. In our study, we utilized the term “LyR” to represent roots, “LyL” to represent leaves, “LyS” to represent stamens, “LyP” to represent pistils, and “LyF” to represent fruits. In total, 43,471 genes were detected in the RNA-seq data across the genome of Longya10. Among all samples showing differential expression levels, 34,497 genes were detected between different organs’ samples, and a heatmap was generated to visualize the gene expression patterns in different organs of Longya10 (Figure 1a). It was observed that gene expression varied across different organs, and there were also differences in expression-regulating genes within specific organs. However, 23,448 genes were found to be commonly expressed across all organs. For further exploration of gene expression in different organs of flax, gene expression comparisons were performed for different parts of Longya10. As shown in the Venn diagram, there were 28,281 expressed genes in LyS, 28,301 in LyP, 28,888 in LyL, 29,321 in LyR, and 29,574 in LyF.

Next, the Venn diagram results for different organs of Longya10 were filtered, and 236, 690, 544, 909, and 1212 genes with organ-specific expression in LyP, LyF, LyL, LyR, and LyS (Figure 1b, Table S1), respectively, were subjected to GO enrichment analysis. Genes with an adjusted False Discovery Rate (FDR) < 1 were annotated using the GO database. These well-annotated sequences were classified into three main categories: Biological Processes (BP), Cellular Components (CC), and Molecular Functions (MF). In the BP category, significant enrichment was observed for GO terms such as cell wall organization or biogenesis (GO:0071554), cell wall organization (GO:0071555), extracellular structure organization (GO:0045229), protein phosphorylation (GO:0006468), and phosphorylation (GO:0016310) (Figure 1c–e,g). However, for roots, enrichment of different processes was observed, including monovalent inorganic cation metabolic process (GO:0044710), oxidation-reduction process (GO:0055114), regulation of RNA biosynthetic process (GO:2001141), transcription, DNA-templated (GO:0006355), regulation of biosynthetic process (GO:0009889), and regulation of macromolecule biosynthetic process (GO:0010556), among others (Figure 1f). In the CC category, significant enrichment was observed for the extracellular encapsulating structure (GO:0016844), cell wall (GO:0016843), and periphery of the cell (GO:0006793) (Figure 1e–g). In the MF category, serine-type peptidase activity (GO:0017171), serine-type endopeptidase activity (GO:0008236), and nucleic acid binding (GO:0003677) were the most enriched GO terms in pistils and fruits. In leaves and stamens, protein kinase activity (GO:0004672), phosphotransferase activity (GO:0016773), alcohol as acceptor, kinase activity (GO:0016301), and others were significantly enriched (Figure 1c,e,g). Additionally, common enrichment of oxidoreductase activity (GO:0016491) was observed in roots and leaves (Figure 1d,f), while heme binding (GO:0020037) and tetrapyrrole binding (GO:0046906) were significantly enriched in roots (Figure 1f).

### 2.2. Gene Expression Profiles and Identification of Differentially Expressed Genes in Various Tissues and Organs of Flax Variety Longya10

**Figure 2:**
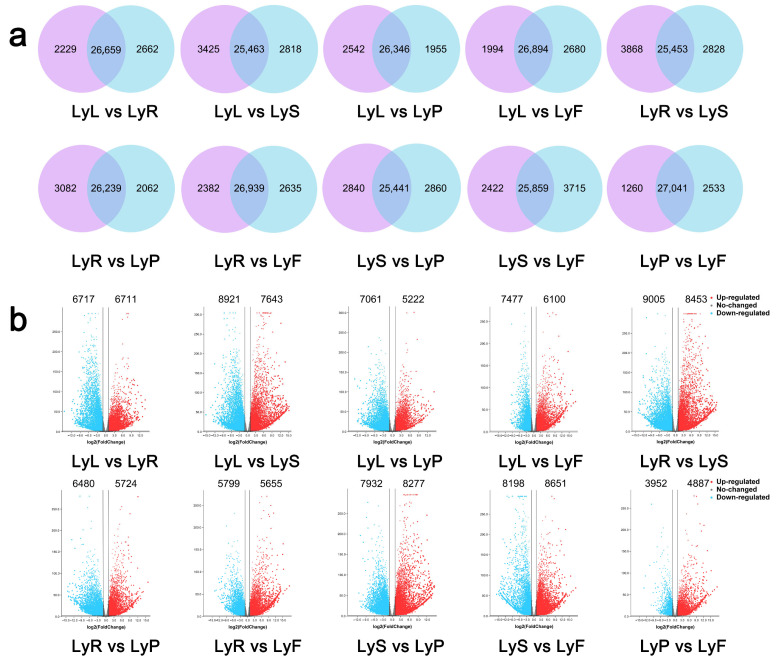
(**a**) Venn diagram representing common and specific gene expression under comparison of different organs of Longya10 flax. (**b**) Volcano plot of differentially expressed gene profiles under different organ comparisons of Longya10 flax. Up-regulated DEGs are marked with red dots and down-regulated DEGs are marked with blue dots. The abbreviations LyR, LyL, LyS, LyP, and LyF represent Longya10 flax roots, leaves, stamens, pistils, and fruits, respectively.

To compare gene expression in different parts of the experimental variety (Figure 2a), we observed that 2662 genes (8.43%) were differentially expressed in roots and 2229 genes (7.06%) were differentially expressed in leaves (LyL vs. LyR, 31,550), while 31,550 genes were commonly expressed in both root and leaf tissues. Furthermore, comparing stamens and leaves (LyL vs. LyS, 31,706), we found that 2818 genes (8.89%) were specifically expressed in stamens and 3425 genes (10.80%) were specifically expressed in leaves, with 31,706 genes expressed in both stamen and leaf tissues. Comparing pistils and leaves (LyL vs. LyP, 30,843), we observed that 1955 genes (6.34%) were specifically expressed in pistils and 2542 genes (8.24%) were specifically expressed in leaves, with 30,843 genes expressed in both pistil and leaf tissues. Additionally, in the comparison of fruits with leaves (LyL vs. LyF, 31,568), we found that 2680 genes (8.49%) were specifically expressed in fruits and 1994 genes (6.32%) were specifically expressed in leaves, with 26,894 genes expressed in both fruit and leaf tissues. Further comparing the gene expression between stamens and roots (LyR vs. LyS, 32,149), we found 2828 genes (8.80%) specifically expressed in stamens and 3868 genes (12.03%) specifically expressed in roots, with 25,453 genes expressed in both stamen and root tissues. In the comparison of pistils and roots (LyR vs. LyP, 31,383), 2062 genes (6.57%) were specifically expressed in pistils and 3082 genes (9.82%) were specifically expressed in roots, with 26,239 genes expressed in both pistil and root tissues. Comparing fruits with roots (LyR vs. LyF, 31,956), we found 2635 genes (8.24%) specifically expressed in fruits and 2382 genes (7.45%) specifically expressed in flax roots, with 26,939 genes expressed in both fruit and root tissues. In the comparison of stamens and pistils (LyS vs. LyP, 31,141), 2840 genes (9.12%) were specifically expressed in stamens and 2860 genes (9.18%) were specifically expressed in pistils, while 25,441 genes were commonly expressed in both stamens and pistils. Lastly, comparing fruit and stamens (LyS vs. LyF, 31,996), we found 3715 genes (11.61%) specifically expressed in fruits and 2422 genes (7.57%) specifically expressed in stamens, with 25,859 genes expressed in both fruit and stamen tissues, indicating a more significant influence of stamens and pistils on fruit formation. Comparing fruit with pistils (LyP vs. LyF, 30,834), 2533 genes (8.21%) were specifically expressed in fruits, while only 1260 genes (4.09%) were specifically expressed in pistils, with 27,041 genes expressed in both fruit and pistil tissues.

To investigate DEGs in various tissues and organs of the flax variety Longya10, we analyzed the DEGs in the stamens, pistils, roots, leaves, and fruits (Figure 2b). We detected many DEGs in each comparison, and their up-regulation and down-regulation patterns are shown in the volcano plots. First, in the comparison of roots and leaves (LyL vs. LyR), we detected 13,428 DEGs, with 6711 genes (49.978%) up-regulated and 6717 genes (50.022%) down-regulated. Next, in the comparison of stamens and pistils (LyS vs. LyP), we observed 16,209 DEGs, with 8277 genes (51.064%) up-regulated and 7932 genes (48.936%) down-regulated. Additionally, we compared pistils with roots and leaves separately. In comparing pistils with roots (LyR vs. LyP) and leaves (LyL vs. LyP), we found 12,204 and 12,283 DEGs, respectively. Comparing roots with pistils, 5724 genes (46.903%) were up-regulated and 6480 genes (53.097%) were down-regulated, while in comparing leaves with pistils, 5222 genes (42.514%) were up-regulated and 7061 genes (57.486%) were down-regulated. These comparisons indicated significant down-regulation of DEGs in pistils compared to roots and leaves. Furthermore, in comparing stamens with roots (LyR vs. LyS) and leaves (LyL vs. LyS), we observed 17,458 DEGs in the roots, with 8453 genes up-regulated and 9005 genes down-regulated. In the leaves, there were 16,564 DEGs, with 7643 genes up-regulated and 8921 genes down-regulated. Notably, significant down-regulation of DEGs was observed only in the leaves in comparing stamens with roots and leaves. In comparing fruits with stamens and roots, no significant differences were observed in the up-regulation or down-regulation of DEGs. However, in comparing fruits with pistils (LyP vs. LyF), 4887 genes (55.289% of the total 8839 DEGs) were up-regulated, showing a significant up-regulation trend. In comparing fruits with leaves, there were 13,577 DEGs, with 7477 genes (55.071%) down-regulated, indicating a significant down-regulation trend.

### 2.3. In-Depth Investigation of Differential Gene Expression Profiles in Different Tissues and Organs of Flax 

**Figure 3:**
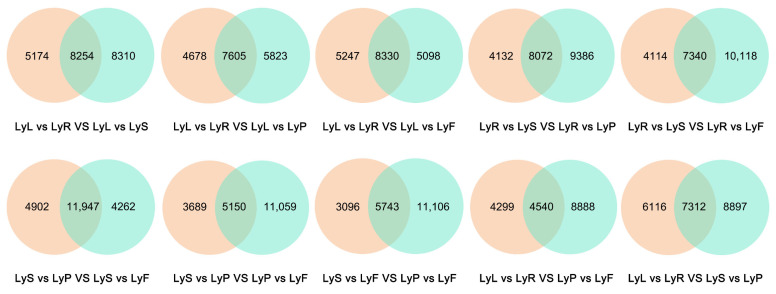
Venn diagrams representing the gene expression of common and specific DEGs in different organs of Longya10 flax after a two-by-two comparison. The abbreviations LyR, LyL, LyS, LyP, and LyF represent the root, leaf, stamen, pistil, and fruit of Longya10 flax, respectively.

In the comparative analysis of different tissues and organs of flax variety Longya10, we summarized the DEGs obtained from pairwise comparisons and further compared them in this study. The comparison of roots and leaves was used as a reference in relation to other comparisons with DEGs in different groups (Figure 3). We have also conducted additional comparisons, which are included in the Supplementary Materials (Figure S1). In the LyL vs. LyR VS LyL vs. LyS comparisons, 5174 genes (23.80%) showed specific expression in the roots and leaves group, while 8310 genes (38.23%) showed specific expression in the leaves and stamens group. In the comparisons of LyL vs. LyR VS LyL vs. LyP, 4678 genes (25.84%) showed specific expression in the roots and leaves group, while 5823 genes (32.16%) showed specific expression in the leaves and pistils group. In the comparisons of LyL vs. LyR VS LyL vs. LyF, 5247 genes (28.10%) showed specific expression in the roots and leaves group, while 5098 genes (27.30%) showed specific expression in the leaves and fruits group. In the comparisons of LyR vs. LyS VS LyR vs. LyP, 4132 genes (19.14%) showed specific expression in the roots and stamens group, while 9386 genes (43.47%) showed specific expression in the roots and pistils group. In the LyR vs. LyS VS LyR vs. LyF comparisons, 4114 genes (19.07%) showed specific expression in the roots and stamens group, while 10,118 genes (46.90%) showed specific expression in the roots and fruits group. In the comparisons of LyS vs. LyP VS LyS vs. LyF, 4902 genes (23.22%) showed specific expression in the stamens and pistils group, while 4262 genes (20.19%) showed specific expression in the stamens and fruits group. In the comparisons of LyS vs. LyP VS LyP vs. LyF, 3689 genes (18.54%) showed specific expression in the stamens and pistils group, while 11,059 genes (55.58%) showed specific expression in the pistils and fruits group. In the comparisons of LyS vs. LyF VS LyP vs. LyF, 3096 genes (15.52%) showed specific expression in the stamens and fruits group, while 11,106 genes (55.68%) showed specific expression in the pistils and fruits group. In the LyL vs. LyR VS LyP vs. LyF comparisons, 4299 genes (24.25%) showed specific expression in the roots and leaves group, while 8888 genes (50.14%) showed specific expression in the pistils and fruits group. In the LyL vs. LyR VS LyS vs. LyP comparisons, 6116 genes (27.40%) showed specific expression in the roots and leaves group, while 8897 genes (39.58%) showed specific expression in the stamens and pistils group.

### 2.4. Annotation and Functional Classification of DEGs in Different Tissues and Organs of Flax Variety Longya10

**Figure 4:**
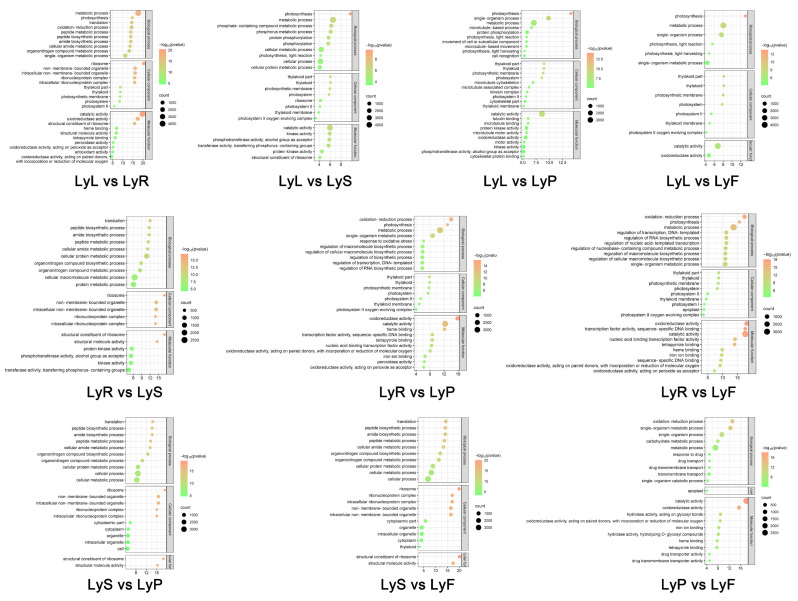
GO classification and enrichment analysis of Differentially Expressed Genes (DEGs) among Longya10 roots, leaves, stamens, pistils, and fruits. The acronyms LyR, LyL, LyS, LyP, and LyF represent Longya10 flax roots, leaves, stamens, pistils, and fruits, respectively.

To comprehensively investigate gene functionality, we performed GO enrichment analysis for the DEGs in various tissues and organs of the flax variety Longya10. After selecting DEGs with significant enrichment (FDR < 0.05), we annotated them using the GO database (Figure 4, Table S2). The analysis revealed the following findings regarding the enriched pathways and functions: In the biological process category, pathways related to metabolism (GO:0008152), single-organism processes (GO:0044699), single-organism metabolic processes (GO:0044710), and oxidation-reduction processes (GO:0055114) were significantly enriched in various organs. Furthermore, DEGs involved in cell processes (GO:0009987), cellular protein metabolic processes (GO:0044267), and cellular metabolic processes (GO:0044237) were specifically enriched in stamens compared to other organs, indicating their potential involvement in pollen synthesis and metabolism in stamens. Comparisons between stamens and other organs revealed significant enrichment of several BPs, including peptide biosynthetic processes (GO:0043043), cellular amide metabolic processes (GO:0043603), organic nitrogen compound biosynthetic processes (GO:1901566), and organic nitrogen compound metabolic processes (GO:1901564). Additionally, the comparison between leaves and other organs showed significant enrichment of the photosynthesis pathway (GO:0015979). In the cellular component (CC) category, a few DEGs were enriched in GO terms related to photosystems (GO:0009521), thylakoid membranes (GO:0034357), and plastids (GO:0009579). Comparisons between stamens and other organs also revealed enrichment of ribosomes (GO:0005840), organelles (GO:0043226), and non-membrane-bound organelles (GO:0043232). In the molecular function (MF) category, catalytic activity (GO:0020037) and oxidoreductase activity (GO:0016491) were the most enriched GO terms for the DEGs. Other enriched functions included heme binding (GO:0003824), tetrapyrrole binding (GO:0016684), protein kinase activity (GO:0004672), kinase activity (GO:0016301), and transferase activity (GO:0003735). These results provide valuable insights into the functional classification of DEGs in different tissues and organs of the flax variety Longya10.

### 2.5. Identification of AP2 Transcription Factors and Analysis of Expression Patterns of Their Family Genes in Different Tissues and Organs of Flax 

**Figure 5:**
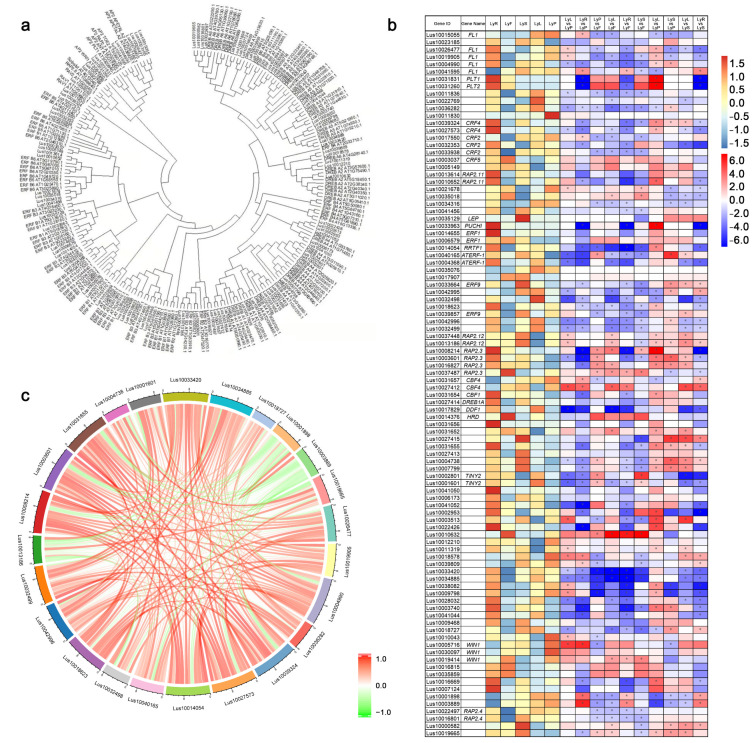
(**a**) Phylogenetic tree clustering of AP2 family genes from *Arabidopsis* and Longya10. (**b**) Heatmap of 96 AP2 family genes in different tissues and comparison groups of Longya10. (**c**) Screening 24 genes with greater than 7 groups as DEGs in different tissue comparison groups of Longya10 and making a correlation coefficient chord plot. The acronyms LyR, LyL, LyS, LyP, and LyF represent Longya10 flax roots, leaves, stamens, pistils, and fruits, respectively. The yellow color represents the expression levels of genes (in log2-transformed read counts per million), while the white color indicates the fold change (log2 value) of the DEGs. Statistical significance is denoted by asterisks (*) with *p* < 0.05.

Transcription factors play an important role in regulating the transcription process of plant genomes [19,36]. Variations in the expression of AP2 family transcription factor genes are associated with adaptation to environmental changes and the growth and development of the plant’s own organs, enabling them to play a role in plant stress tolerance and organ differentiation [36]. We performed phylogenetic tree clustering analysis of genes from a family of 226 AP2 transcription factors in *Arabidopsis thaliana* and Longya10 flax and screened 96 AP2-associated transcription factor genes that are homologous in Longya10 (Figure 5a). The overall expression analysis by heatmap showed that root gene expression was significantly up-regulated, while these genes were significantly down-regulated in fruits (Figure 5b, Table S3). Some of the AP2 transcription factor family genes also showed different up- or down-regulated regulatory expression responses of DEGs in different comparisons. As a result, some of the genes showed a significant down-regulation trend in comparing 10 groups of different tissues in which they were expressed (Figure 5b). Genes such as *Lus10011836*, *Lus10036282*, *Lus10014054*, *Lus10040165*, *Lus10004368*, *Lus10032498*, *Lus10017829*, *Lus10033420*, *Lus10034885*, and *Lus10028032* were significantly down-regulated across comparisons; however, genes such as *Lus1006827*, *Lus10037487*, *Lus10027412*, *Lus10010632*, *Lus10000582*, and *Lus10019665* were significantly up-regulated across comparisons. After that, we screened 96 genes that were greater than 7 times as DEGs in different organ comparison groups of Longya10 and obtained 24 genes (*Lus10026477*, *Lus10019905*, *Lus10004990*, *Lus10036282*, *Lus10039324*, *Lus10027573*, *Lus10014054*, *Lus10040165*, *Lus10032498*, *Lus10018623*, *Lus10042996*, *Lus10032499*, *Lus10013186*, *Lus10008214*, *Lus10003601*, *Lus10031655*, *Lus10004738*, *Lus10001601*, *Lus10033420*, *Lus10034885*, *Lus10018727*, *Lus10001898*, *Lus10003889*, *Lus10019665*), and their expressions in different tissues were made into correlation coefficient chordal plots (Figure 5c, Table S4). The red curve represents a positive correlation, and the green curve represents a negative correlation. From the graph, the results show some genes (*Lus10004990*, *Lus10039324*, *Lus10027573*, *Lus10014054*, *Lus10018623*, *Lus10008214*, *Lus10003601*, *Lus10031655*, *Lus10033420*, *Lus10034885*) are significantly positively correlated with other genes, while some genes (*Lus10013186*, *Lus10001898*, *Lus10003889*, *Lus10019665*) are significantly negatively correlated with other genes (Figure 5c).

### 2.6. qRT-PCR Validation of RNA-Seq

**Figure 6:**
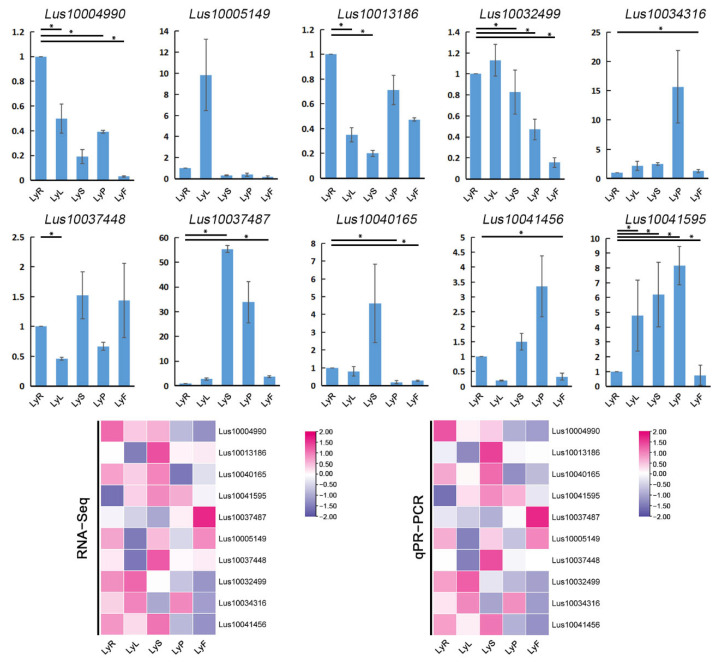
(**a**) The results of qRT-PCR to amplify the expression of ten genes. (**b**) The comparison results of RNA-seq and qRT-PCR. The acronyms LyR, LyL, LyS, LyP, and LyF are root, leaf, stamen, pistil, and fruit of Longya10 flax, respectively. Statistical significance is denoted by asterisks (*) with *p* < 0.05.

We performed qRT-PCR validation on the RNA-seq analysis results of various tissues of the flax variety Longya10, including roots, leaves, pistils, stamens, and fruits (Figure 6). After screening primers, we selected 10 genes (*Lus10004990*, *Lus10013186*, *Lus10040165*, etc.). These genes belong to four subfamilies, AP2 APETAL A2, ERF B2, ERF B3, and ERF B5, and function in integrating enzymatic DNA binding superfamily proteins, ethylene response element binding factors, and cytokinin response factors. Among them, *Lus10004990*, *Lus10013186*, *Lus10040165*, *Lus10041595*, *Lus10037487*, *Lus10037448*, *Lus10032499*, and *Lus10034316* are more commonly differentially expressed genes in the comparisons of various tissues of Longya10. *Lus10004990*, *Lus10013186*, *Lus10040165*, and *Lus10032499* are the most differentially expressed genes in different comparisons and are DEGs in comparisons between leaves and pistils, fruits and stamens, and fruits and pistils. We also found that there are more differentially expressed genes in comparisons between fruits and roots, leaves, and stamens, and genes *Lus10004990*, *Lus10040165*, *Lus10037487*, and *Lus10032499* are DEGs in these comparisons. However, in all comparisons, only gene *Lus10005149* is not a differentially expressed gene. The qRT-PCR validation results confirmed that the expression levels of the selected 10 genes were consistent with the expression levels measured by RNA-seq.

## 3. Discussion

The differences among various flax tissues are related to morphological variations in key areas and differences in nutrient consumption during different developmental stages. Different genes may also play important roles in different tissues and organs [37,38]. This study compared the gene expression of flax roots, leaves, stamens, pistils, and fruits in Longya10. We selected genes commonly expressed in different tissue comparisons and genes completely expressed in all tissues. By analyzing these genes’ functions and molecular pathways comprehensively, we have provided references for understanding the specific regulatory mechanisms of different organs in Longya10 flax. Although roots and leaves at different growth stages may have different gene expression patterns and functions, in this experiment, we chose to analyze the roots and leaves of flax materials grown at an early stage (three weeks) for gene analysis. This is because gene expression is relatively active during the early stages of plant growth and development, where leaves and roots undergo significant physiological and morphological changes [39]. Comparing roots and leaves at the same growth stage can reduce the interference caused by differences in growth stages, and at this stage, roots and leaves are relatively easy to obtain, and their tissue structure and physiological status are relatively stable.

Our GO analysis results showed a significant enrichment of metabolic pathways in different organ comparisons of Longya10. Specifically, we found that genes and metabolites related to photosynthesis were significantly enriched in leaf tissues, while genes associated with organic synthesis and metabolism were significantly enriched in fruit tissues. The flax roots showed enrichment in nutrient absorption, transport, regulation, and oxidation-reduction pathways. Additionally, pathways related to metabolism and acylamine were significantly enriched in roots, leaves, and fruits, indicating their important role in plant stress resistance [40,41,42]. The pathways related to developing flax pistils and stamens showed significant enrichment in kinase activity, oxidoreductase activity, and catalytic activity. Overall, this study provides valuable insights into the tissue-specific regulatory mechanisms of different organs in Longya10 flax. The identified genes and enriched pathways shed light on the underlying molecular processes and could potentially contribute to improving flax breeding and cultivation strategies.

In this study, genes such as *Lus10040165*, *Lus10004368*, *Lus10033664*, *Lus10039857*, *Lus10031652*, *Lus10022426*, and *Lus10016669* showed significant differential expression in various organ comparisons, with the expression being higher in roots than in leaves but not with low expression levels. However, these genes were all downregulated in comparing roots and fruits. *Lus10022426* and *Lus10016669* were upregulated in the comparisons between roots and leaves but downregulated in the comparisons with pistils, fruits, and stamens. Genes such as *Lus10042995*, *Lus10032498*, *Lus10042996*, and *Lus10032499* had higher expression levels in leaves than in roots, which is different from previous reports. *OsEREBP1* is a phosphorylated transcription factor reported in rice, which, together with BWMK1, transiently co-expressed in *Arabidopsis* protoplasts and increased the expression of a beta-glucosidase reporter gene driven by GCC elements, possibly participating in the transcription activation of defense signal pathways [43]. We found that the expression of the gene annotated as an oxidation-reduction responsive transcription factor, *Lus10014054*, was significantly upregulated in roots. Genes such as *Lus10037448*, *Lus10013186*, *Lus10008214*, *Lus10003601*, *Lus10016827*, *Lus10037487*, *Lus10018578*, and *Lus10039809*, which encode ethylene response element-binding proteins and integrase-type DNA binding superfamily proteins, may be related to regulatory genes with the same function as rice mitogen-activated protein kinase BWMK1 [43]. *Lus10037448* was only upregulated in leaf comparisons with other tissues. *Lus10013186* was upregulated in all comparisons that showed differential expression, except for the comparisons with fruits and stamens, where it was downregulated. Finally, *Lus10014054* was upregulated in comparisons between roots and leaves but was generally downregulated in all other comparisons where it was identified as a differentially expressed gene. The AP2 ethylene response transcription factor PLT is expressed in various organs such as roots, seedlings, and flowers. It is specifically detected in the distal root meristem of plant roots, and it regulates root polar auxin transport by modulating the distribution of PIN genes [44,45]. In this study, we found that *Lus10031831* and *Lus10031260* are significantly more highly expressed in roots compared to other tissues. In previous studies, BBM was discovered by in vitro induction of immature pollen grains in rapeseed, showing similarity to the AP2/ERF family of transcription factors, which are preferentially expressed in developing embryos and seeds and promote cell proliferation, differentiation and morphogenesis [46,47]. In our study, we found that *Lus10015055*, *Lus10026477*, *Lus10019905*, *Lus10004990*, and *Lus10041595* show significantly increased expression in pistils, stamens, leaves, and roots, but the expression level in fruits remains stable, which differs from previous research. RAV1 is a novel DNA-binding protein with two distinct DNA-binding domains. Overexpression of RAV1 in *Arabidopsis* leads to delayed lateral root and gynoecium development, suggesting that RAV1 may function as a negative regulatory component in growth and development [48]. The RAV1 subfamily gene *Lus10036282*, unique in the phylogenetic tree, was identified as a DEG in various organ comparisons, with significantly higher expression in leaves compared to other tissues. In the ERF VII subfamily, RAP2 genes encode two classes of proteins, which show differential expression in flowers, leaves, inflorescence stems, and roots [49]. The transcription factor RAP2.11, by regulating high-affinity K+ uptake transporter AtHAK5 and other components of the low-potassium signaling pathway, is identified as a component involved in the response to low potassium stress, with many genes being upregulated when RAP2.11 is overexpressed in roots [50]. Under low oxygen concentration conditions, such as during flooding, RAP2.12 is released from the plasma membrane and accumulates in the nucleus to activate gene expression in hypoxia adaptation [51]. Meanwhile, RAP2.2 partially overlaps with RAP2.12 and regulates the expression of low-oxygen stress-related genes including ADH1 and PDC1, and it also regulates the expression of genes associated with carbohydrate metabolism and ethylene synthesis [52,53]. In this study, the expression level of the *Lus10010652* gene was significantly increased in roots, and it was identified as a DEG in comparisons between flax roots and other tissues, with a significantly upregulated expression level in comparison with leaves and stamens. *Lus10037448*, *Lus10013186*, *Lus10008214*, *Lus10003601*, *Lus10016827*, *Lus10037487*, *Lus10022497*, and *Lus10016801* genes displayed significantly increased expression levels in various tissues, and the number of upregulated genes was greater than that of the downregulated genes in comparisons between these tissues. The expression levels of *Lus10022497* and *Lus10016801* genes were significantly lower in fruits than in other tissues, and both of them were downregulated genes in comparisons between fruits and leaves, roots, pistils, and stamens. Our results support the phenomenon of tissue-specific gene expression during plant growth and development [54,55].

In addition to the ethylene signaling pathway, some unknown members of the *Arabidopsis* AP2 gene family have been named cytokinin response factors (CRFs) due to their close correlation with the cell division and cytokinin signal transduction pathway [56]. They may act as transcriptional activators that regulate gene expression in response to stress factors and stress signal transduction pathways and participate in developing cotyledons and leaves [56]. In our data, *Lus10039324*, *Lus10027573*, *Lus10017550*, *Lus10032353*, and *Lus10033938* genes showed high expression levels in roots, leaves, and stamens, among which the downregulated genes occupied a larger proportion in DEG comparisons. *Lus10032353* and *Lus10033938* were downregulated genes in all four DEG comparisons, and *Lus10033938* was downregulated in comparisons between fruits and other tissues, suggesting that cytokinin regulatory signals may play a role in the growth and development of tissues except for fruits.

Furthermore, previous studies have shown that the *Arabidopsis* PUCHI gene, encoding the AP2/ethylene response element binding protein transcription factor, acts downstream of the auxin signal transduction pathway and participates in regulating the cell division pattern during early lateral root primordium development to promote lateral root formation [57]. In this study, we identified *Lus10033963* as a PUCHI regulator, which was significantly upregulated in roots and consistent with previous research. However, in addition to being significantly upregulated as a DEG in comparing roots and leaves, it was significantly downregulated in comparisons between roots and pistils, stamens, and fruits.

The *Arabidopsis* CBF/DREB1 transcriptional activators are key regulatory factors in gene expression during low-temperature adaptation signaling [58]. The overexpression of the CBF4 gene in *Arabidopsis* leads to the activation of C-repeat/dehydration responsive elements (CRT/DRE) downstream genes involved in cold and drought adaptation, thereby conferring greater tolerance to low temperature and drought stress. Furthermore, the specificity of CBF1 gene induction is primarily induced by cold stress rather than osmotic stress in leaves, roots, and stems [58,59,60]. In this study, we found that the expression level of *Lus10031657* was the lowest in roots and significantly up-regulated in comparisons between roots and pistils as well as stamens. Interestingly, the gene *Lus10027412* had the lowest expression levels in roots and leaves, but it was significantly up-regulated in comparisons between roots and pistils, stamens, and fruits. On the other hand, *Lus10031654* showed significant up-regulation in roots, but it was significantly down-regulated in comparisons between roots and pistils as well as fruits, while showing up-regulation in comparison with leaves.

In *Arabidopsis*, WIN1 is an ethylene-responsive element binding transcription factor that activates overexpressed wax deposition in plants. It is detected in very young, closed sepal buds and subsequently expressed in sepal veins and epidermis but not in stamens [61,62]. In our data, *Lus10005716* showed a significant increase in expression in pistils. It was significantly up-regulated in comparisons with roots and leaves as DEGs but down-regulated in comparisons with fruits and stamens. *Lus10019414* showed a significant increase in expression in pistils and fruits and was up-regulated in comparisons with roots, leaves, and stamens as DEGs. Both *Lus10005716* and *Lus10019414* were lowly expressed in stamens, consistent with previous studies. 

The reported transcriptome profiles of flax stems revealed key genes involved in lignin biosynthesis, which is important for fiber quality in flax. It was utilized to improve flax crops’ fiber quality and yield [63,64]. In the previous study, we identified differentially expressed genes and enriched pathways related to stress response, hormone signaling, and physiological characteristics, which can be applied to develop drought-tolerant flax varieties and enhance crop productivity under water-limited conditions [65]. Furthermore, in the previous report, the gene expression profiles of flax seeds during development and identified genes involved in fatty acid biosynthesis and seed storage, which are used to enhance the oil content and quality of flax seeds, are important for various industrial applications [66,67]. These findings contribute to understanding the molecular processes underlying flax gene expression and provide valuable resources for improving flax breeding and cultivation strategies. Our results also targeted specific genes and pathways identified by transcriptome profiles, giving the information to the breeders and researchers to develop new flax varieties with improved traits such as fiber quality, stress tolerance, and oil content.

## 4. Materials and Methods

### 4.1. Plant Materials and Treatment

For this experiment, the oilseed flax Longya10 variety was chosen as the material. XinJiang University supported the flax seeds of Longya10 variety. Fully mature and uniformly sized Longya10 seeds were selected and subjected to sterilization in 1% sodium hypochlorite solution for 2 min. The seeds were thoroughly washed with distilled water and then transferred to a greenhouse under a 26/18 °C 16/8 h light/dark regime. The treated seeds were planted in soil (vermiculite 1:1 mix) until the flax seedlings grew to 8–10 cm in about three weeks, then carefully removed from the soil and rinsed. The roots and leaves of uniform and well-developed Longya10 seedlings were collected and immediately frozen in liquid nitrogen. Additionally, the plants were waited to grow until the flowering stage (about 12 weeks). Male and female reproductive organs (stamens and pistils) were collected from Longya10 plants and frozen in liquid nitrogen. Meanwhile, flax fruits were collected and immediately frozen in liquid nitrogen. Samples were stored at −80 °C before RNA extraction. Three biological replicates were collected for each tissue type, including stamens, pistils, leaves, roots, and fruits of the Longya10 variety.

### 4.2. RNA Extraction and Sequencing

The collected Longya10 flax tissue samples were used to extract total RNA using the Plant Total RNA Extraction Kit. To ensure the reliability of the subsequent experiments, RNA quality was analyzed by agarose gel electrophoresis, following the previously reported method of RNA extraction and cDNA library construction [68]. Each sample was analyzed with three biological replicates. The high-throughput sequencing platform (Illumina HiSeq 2000, San Diego, CA, USA) was employed to sequence the RNA after passing the quality control. We removed the adaptor, poly-A, and low-quality reads and obtained > 4 Gb clean data with pairs of 150 bp reads of each sample (Table S5). Both Q20 and Q30 were greater than 92%.

### 4.3. Differential Expression Gene Screening and GO Enrichment Analysis

DESeq2 software (version 1.20.0) was utilized to perform comparisons of different flax tissues and conduct differential expression analyses between combinations, with each group having three biological replicates [69]. To control for false positives, *p*-values were adjusted using the Benjamini and Hochberg method with a calibrated threshold of *p* < 0.01. DEGs were screened based on the criteria that |log2foldchange| > 1 and the False Discovery Rate (FDR) < 0.05. Following the DEG screening, comparative analyses between the different treated materials were conducted. Molecular functional class Gene Ontology (GO) terms were annotated using the online agriGOv2 platform (http://systemsbiology.cau.edu.cn/agriGOv2/index.php (accessed on 6 May 2023)) [69]. Additionally, GO enrichment analysis of the differentially expressed genes was performed using the microbioinformatics platform (http://www.bioinformatics.com.cn/ (accessed on 13 May 2023)). Heatmaps were generated from the experimental data using TBTOOLs and RStudio for data visualization. Furthermore, selected genes, such as flax AP2, were mapped to *Arabidopsis* genes using MEGA software (version 11.0.10) to construct phylogenetic trees. Additionally, chordal plots were produced using the microbioinformatics platform.

### 4.4. Data Processing

The experimental data in this study involved different flax tissue treatments, and each was replicated independently three times. Statistical analysis of the experimental data was performed using Microsoft Excel. Analysis of Variance was employed to determine the significance of differences between the experimental treatments. In this analysis, the significance levels were set at *p* < 0.05 to indicate statistically significant differences between treatments and *p* < 0.01 to indicate highly significant differences.

### 4.5. qRT-PCR Validation

To verify the RNA-seq data’s reliability and quantify the expression of an AP2-ERF transcription factor in flax under saline stress, the SYBR Green I PCR master mix kit (TaKaRa, Maebashi, Japan) was used in the qRT-PCR reactions as in our previous reports [70]. Gene-specific primers (Table S6) were downloaded from qPrimerDB [71].

## 5. Conclusions

In this study, we investigated the changes in gene expression across various tissues and organs, including roots, leaves, stamens, pistils, and fruits, in the flax cultivar Longya10. We identified genes commonly and specifically expressed in different tissues and organs and annotated them into various pathways associated with organ comparisons. These findings offer insights into the regulatory networks governing organ-specific gene expression in flax and serve as a valuable reference for flax plant cultivation. Furthermore, we focused on identifying and screening different transcription factors within the AP2 transcription factor family. This approach enables us to explore the functions of these transcription factors and unravel the molecular mechanisms through which they regulate various biological pathways in flax tissues. Next, further research is needed on the AP2 transcription factors in flax, particularly regarding their response to different stress conditions. This will involve investigating the expression patterns of these genes in response to various stressors, such as drought, salinity, and pathogen infection. By understanding how these transcription factors respond to stress, we can better understand the regulatory mechanisms underlying stress tolerance in flax. Importantly, our study revealed significant expression differences among different tissues and organs of flax. This information provides valuable insights for future crop breeding efforts to develop flax varieties that can adapt to changing environmental conditions.

## Data Availability

The datasets generated and analyzed in this study are available at [PRJNA1002756] https://www.ncbi.nlm.nih.gov/sra/PRJNA1002756 (accessed on 7 August 2023).

## Supplementary Materials

The following supporting information can be downloaded at: https://www.mdpi.com/article/10.3390/plants12183260/s1, Table S1: The overview of RNA-seq data; Table S2: The data of different tissue-specific GO analyses; Table S3: The data from different organizations comparing GO analyses; Table S4: The data of AP2-related gene expression analysis; Table S5: The data from the comparative analysis of AP2-related genes expressed as DEGs greater than seven times; Table S6: The list of primers for qRT-PCR. Figure S1: The gene expression of common and specific DEGs in different organs of Longya10 flax after a two-by-two comparison.

## References

[1] Dash J., Naik B., Mohapatra U. (2017). Linseed: A Valuable Crop Plant. Int. J. Adv. Res..

[2] Dikshit A., Gao C., Small C., Hales K., Hales D.B. (2016). Flaxseed and its components differentially affect estrogen targets in pre-neoplastic hen ovaries. J. Steroid Biochem. Mol. Biol..

[3] Pal P., Hales K., Petrik J., Hales D.B. (2019). Pro-apoptotic and anti-angiogenic actions of 2-methoxyestradiol and docosahexaenoic acid, the biologically derived active compounds from flaxseed diet, in preventing ovarian cancer. J. Ovarian Res..

[4] Jiang X., Wang X., Zhou S. (2022). Effect of flaxseed marc flour on high-yield wheat bread production: Comparison in baking, staling, antioxidant and digestion properties. LWT.

[5] Koksharov S., Aleeva S., Lepilova O., Kalinin E., Kornilova N. (2023). How to transform lignin into a useful component of flax fiber for composite materials. Ind. Crops Prod..

[6] Bar M., Grégoire M., Khan S.U., De Luycker E., Ouagne P. (2022). Studies on Classically Harvested Linseed Flax Fibers for Bio-composite Reinforcement and Textile Applications. J. Nat. Fibers.

[7] Baladivakar S., Starvin M.S., Raj J.B. (2022). Mechanical and Thermal Characteristics of Hybrid Composites Fortified with Flax, Banyan, and Glass Fibers for Automobile Safety Applications. J. Nat. Fibers.

[8] Öpik H., Rolfe S.A. (2005). Cell growth and differentiation. the Physiology of Flowering Plants.

[9] Lux A., Rost T.L. (2012). Plant root research: The past, the present and the future. Ann. Bot..

[10] Du F., Guan C., Jiao Y. (2018). Molecular Mechanisms of Leaf Morphogenesis. Mol. Plant.

[11] Scott R.J., Spielman M., Dickinson H.G. (2004). Stamen structure and function. Plant Cell.

[12] Palanivelu R., Johnson M.A. (2010). Functional genomics of pollen tube-pistil interactions in Arabidopsis. Biochem. Soc. Trans..

[13] Erbasol Serbes I., Palovaara J., Gross-Hardt R. (2019). Development and function of the flowering plant female gametophyte. Curr. Top Dev. Biol..

[14] Yadegari R., Drews G.N. (2004). Female gametophyte development. Plant Cell.

[15] Yaschenko A.E., Fenech M., Mazzoni-Putman S., Alonso J.M., Stepanova A.N. (2022). Deciphering the molecular basis of tissue-specific gene expression in plants: Can synthetic biology help?. Curr. Opin. Plant Biol..

[16] Guan Y., Li G., Chu Z., Ru Z., Jiang X., Wen Z., Zhang G., Wang Y., Zhang Y., Wei W. (2019). Transcriptome analysis reveals important candidate genes involved in grain-size formation at the stage of grain enlargement in common wheat cultivar “Bainong 4199”. PLoS ONE.

[17] Agarwal P., Arora R., Ray S., Singh A.K., Singh V.P., Takatsuji H., Kapoor S., Tyagi A.K. (2007). Genome-wide identification of C_2_H_2_ zinc-finger gene family in rice and their phylogeny and expression analysis. Plant Mol. Biol..

[18] Xu C., Park S.J., Van Eck J., Lippman Z.B. (2016). Control of inflorescence architecture in tomato by BTB/POZ transcriptional regulators. Genes Dev..

[19] Feng K., Hou X.-L., Xing G.-M., Liu J.-X., Duan A.-Q., Xu Z.-S., Li M.-Y., Zhuang J., Xiong A.-S. (2020). Advances in AP2/ERF super-family transcription factors in plant. Crit. Rev. Biotechnol..

[20] Xie Z., Nolan T.M., Jiang H., Yin Y. (2019). AP2/ERF Transcription Factor Regulatory Networks in Hormone and Abiotic Stress Responses in Arabidopsis. Front. Plant Sci..

[21] Ye B.B., Shang G.D., Pan Y., Xu Z.G., Zhou C.M., Mao Y.B., Bao N., Sun L., Xu T., Wang J.W. (2020). AP2/ERF Transcription Factors Integrate Age and Wound Signals for Root Regeneration. Plant Cell.

[22] Dai X., Wang Y., Zhang W.H. (2016). OsWRKY74, a WRKY transcription factor, modulates tolerance to phosphate starvation in rice. J. Exp. Bot..

[23] Guo Y., Huang R., Duan L., Wang J. (2017). The APETALA2/ethylene-responsive factor transcription factor OsDERF2 negatively modulates drought stress in rice by repressing abscisic acid responsive genes. J. Agric. Sci..

[24] Jiang L., Ma X., Zhao S., Tang Y., Liu F., Gu P., Fu Y., Zhu Z., Cai H., Sun C. (2019). The APETALA2-Like Transcription Factor SUPERNUMERARY BRACT Controls Rice Seed Shattering and Seed Size. Plant Cell.

[25] Zhao Q., Hu R.S., Liu D., Liu X., Wang J., Xiang X.H., Li Y.Y. (2020). The AP2 transcription factor NtERF172 confers drought resistance by modifying NtCAT. Plant Biotechnol. J..

[26] Wang X., Han H., Yan J., Chen F., Wei W. (2015). A New AP2/ERF Transcription Factor from the Oil Plant Jatropha curcas Confers Salt and Drought Tolerance to Transgenic Tobacco. Appl. Biochem. Biotechnol..

[27] Zhang B., Su L., Hu B., Li L. (2018). Expression of AhDREB1, an AP2/ERF Transcription Factor Gene from Peanut, Is Affected by Histone Acetylation and Increases Abscisic Acid Sensitivity and Tolerance to Osmotic Stress in Arabidopsis. Int. J. Mol. Sci..

[28] Najafi S., Sorkheh K., Nasernakhaei F. (2018). Characterization of the APETALA2/Ethylene-responsive factor (AP2/ERF) transcription factor family in sunflower. Sci. Rep..

[29] Fujimoto S.Y., Ohta M., Usui A., Shinshi H., Ohme-Takagi M. (2000). Arabidopsis Ethylene-Responsive Element Binding Factors Act as Transcriptional Activators or Repressors of GCC Box–Mediated Gene Expression. Plant Cell.

[30] Koyama T., Kitajima S., Sato F. (2001). Expression of PR-5d and ERF Genes in Cultured Tobacco Cells and Their NaCl Stress-response. Biosci. Biotechnol. Biochem..

[31] Ohme-Takagi M., Shinshi H. (1995). Ethylene-inducible DNA binding proteins that interact with an ethylene-responsive element. Plant Cell.

[32] Yamamoto S., Suzuki K., Shinshi H. (1999). Elicitor-responsive, ethylene-independent activation of GCC box-mediated transcription that is regulated by both protein phosphorylation and dephosphorylation in cultured tobacco cells. Plant J..

[33] Kitajima S., Koyama T., Ohme-Takagi M., Shinshi H., Sato F. (2000). Characterization of Gene Expression of NsERFs, Transcription Factors of Basic PR Genes from Nicotiana sylvestris. Plant Cell Physiol..

[34] Armengaud P., Breitling R., Amtmann A. (2004). The Potassium-Dependent Transcriptome of Arabidopsis Reveals a Prominent Role of Jasmonic Acid in Nutrient Signaling. Plant Physiol..

[35] Zhai R., Feng Y., Wang H., Zhan X., Shen X., Wu W., Zhang Y., Chen D., Dai G., Yang Z. (2013). Transcriptome analysis of rice root heterosis by RNA-Seq. BMC Genom..

[36] Mizoi J., Shinozaki K., Yamaguchi-Shinozaki K. (2012). AP2/ERF family transcription factors in plant abiotic stress responses. Biochim. Biophys Acta.

[37] Liu R., Guo Z., Lu S. (2021). Genome-Wide Identification and Expression Analysis of the Aux/IAA and Auxin Response Factor Gene Family in Medicago truncatula. Int. J. Mol. Sci..

[38] Nadarajah K., Kumar I.S. (2019). Drought Response in Rice: The miRNA Story. Int. J. Mol. Sci..

[39] Jain M., Nijhawan A., Arora R., Agarwal P., Ray S., Sharma P., Kapoor S., Tyagi A.K., Khurana J.P. (2007). F-box proteins in rice. Genome-wide analysis, classification, temporal and spatial gene expression during panicle and seed development, and regulation by light and abiotic stress. Plant Physiol..

[40] Szecowka M., Heise R., Tohge T., Nunes-Nesi A., Vosloh D., Huege J., Feil R., Lunn J., Nikoloski Z., Stitt M. (2013). Metabolic fluxes in an illuminated Arabidopsis rosette. Plant Cell.

[41] Ishikawa S., Barrero J.M., Takahashi F., Nakagami H., Peck S.C., Gubler F., Shinozaki K., Umezawa T. (2019). Comparative Phosphoproteomic Analysis Reveals a Decay of ABA Signaling in Barley Embryos during After-Ripening. Plant Cell Physiol..

[42] Chen L.M., Zhou X.A., Li W.B., Chang W., Zhou R., Wang C., Sha A.H., Shan Z.H., Zhang C.J., Qiu D.Z. (2013). Genome-wide transcriptional analysis of two soybean genotypes under dehydration and rehydration conditions. BMC Genom..

[43] Cheong Y.H., Moon B.C., Kim J.K., Kim C.Y., Kim M.C., Kim I.H., Park C.Y., Kim J.C., Park B.O., Koo S.C. (2003). BWMK1, a rice mitogen-activated protein kinase, locates in the nucleus and mediates pathogenesis-related gene expression by activation of a transcription factor. Plant Physiol..

[44] Aida M., Beis D., Heidstra R., Willemsen V., Blilou I., Galinha C., Nussaume L., Noh Y.S., Amasino R., Scheres B. (2004). The PLETHORA genes mediate patterning of the Arabidopsis root stem cell niche. Cell.

[45] Blilou I., Xu J., Wildwater M., Willemsen V., Paponov I., Friml J., Heidstra R., Aida M., Palme K., Scheres B. (2005). The PIN auxin efflux facilitator network controls growth and patterning in Arabidopsis roots. Nature.

[46] Boutilier K., Offringa R., Sharma V.K., Kieft H., Ouellet T., Zhang L., Hattori J., Liu C.M., van Lammeren A.A., Miki B.L. (2002). Ectopic expression of BABY BOOM triggers a conversion from vegetative to embryonic growth. Plant Cell.

[47] Wang J., Jiao J., Zhou M., Jin Z., Yu Y., Liang M. (2019). Physiological and Transcriptional Responses of Industrial Rapeseed (Brassica napus) Seedlings to Drought and Salinity Stress. Int. J. Mol. Sci..

[48] Hu Y.X., Wang Y.H., Liu X.F., Li J.Y. (2004). Arabidopsis RAV1 is down-regulated by brassinosteroid and may act as a negative regulator during plant development. Cell Res..

[49] Okamuro J.K., Caster B., Villarroel R., Van Montagu M., Jofuku K.D. (1997). The AP2 domain of APETALA2 defines a large new family of DNA binding proteins in Arabidopsis. Proc. Natl. Acad. Sci. USA.

[50] Kim M.J., Ruzicka D., Shin R., Schachtman D.P. (2012). The Arabidopsis AP2/ERF Transcription Factor RAP2.11 Modulates Plant Response to Low-Potassium Conditions. Mol. Plant.

[51] Licausi F., Kosmacz M., Weits D.A., Giuntoli B., Giorgi F.M., Voesenek L.A.C.J., Perata P., van Dongen J.T. (2011). Oxygen sensing in plants is mediated by an N-end rule pathway for protein destabilization. Nature.

[52] Zhao Y., Wei T., Yin K.Q., Chen Z., Gu H., Qu L.J., Qin G. (2012). Arabidopsis RAP2.2 plays an important role in plant resistance to Botrytis cinerea and ethylene responses. New Phytol..

[53] Hinz M., Wilson I.W., Yang J., Buerstenbinder K., Llewellyn D., Dennis E.S., Sauter M., Dolferus R. (2010). Arabidopsis RAP2.2: An ethylene response transcription factor that is important for hypoxia survival. Plant Physiol..

[54] Yang H., Nukunya K., Ding Q., Thompson B.E. (2022). Tissue-specific transcriptomics reveal functional differences in floral development. Plant Physiol..

[55] Tu Z., Shen Y., Wen S., Liu H., Wei L., Li H. (2021). A Tissue-Specific Landscape of Alternative Polyadenylation, lncRNAs, TFs, and Gene Co-expression Networks in Liriodendron chinense. Front. Plant Sci..

[56] Rashotte A.M., Mason M.G., Hutchison C.E., Ferreira F.J., Schaller G.E., Kieber J.J. (2006). A subset of Arabidopsis AP2 transcription factors mediates cytokinin responses in concert with a two-component pathway. Proc. Natl. Acad. Sci. USA.

[57] Hirota A., Kato T., Fukaki H., Aida M., Tasaka M. (2007). The auxin-regulated AP2/EREBP gene PUCHI is required for morphogenesis in the early lateral root primordium of Arabidopsis. Plant Cell.

[58] Sakuma Y., Liu Q., Dubouzet J.G., Abe H., Shinozaki K., Yamaguchi-Shinozaki K. (2002). DNA-binding specificity of the ERF/AP2 domain of Arabidopsis DREBs, transcription factors involved in dehydration- and cold-inducible gene expression. Biochem. Biophys Res. Commun..

[59] Haake V., Cook D., Riechmann J.L., Pineda O., Thomashow M.F., Zhang J.Z. (2002). Transcription factor CBF4 is a regulator of drought adaptation in Arabidopsis. Plant Physiol..

[60] Shinwari Z.K., Nakashima K., Miura S., Kasuga M., Seki M., Yamaguchi-Shinozaki K., Shinozaki K. (1998). An Arabidopsis Gene Family Encoding DRE/CRT Binding Proteins Involved in Low-Temperature-Responsive Gene Expression. Biochem. Biophys. Res. Commun..

[61] Broun P., Poindexter P., Osborne E., Jiang C.-Z., Riechmann J.L. (2004). WIN1, a transcriptional activator of epidermal wax accumulation in Arabidopsis. Proc. Natl. Acad. Sci. USA.

[62] Aharoni A., Dixit S., Jetter R., Thoenes E., van Arkel G., Pereira A. (2004). The SHINE clade of AP2 domain transcription factors activates wax biosynthesis, alters cuticle properties, and confers drought tolerance when overexpressed in Arabidopsis. Plant Cell.

[63] Goudenhooft C., Bourmaud A., Baley C. (2019). Flax (*Linum usitatissimum* L.) Fibers for Composite Reinforcement: Exploring the Link between Plant Growth, Cell Walls Development, and Fiber Properties. Front. Plant Sci..

[64] Povkhova L.V., Melnikova N.V., Rozhmina T.A., Novakovskiy R.O., Pushkova E.N., Dvorianinova E.M., Zhuchenko A.A., Kamionskaya A.M., Krasnov G.S., Dmitriev A.A. (2021). Genes Associated with the Flax Plant Type (Oil or Fiber) Identified Based on Genome and Transcriptome Sequencing Data. Plants.

[65] Wang N., Qi F., Wang F., Lin Y., Xiaoyang C., Peng Z., Zhang B., Qi X., Deyholos M.K., Zhang J. (2023). Evaluation of Differentially Expressed Genes in Leaves vs. Roots Subjected to Drought Stress in Flax (*Linum usitatissimum* L.). Int. J. Mol. Sci..

[66] Dmitriev A.A., Kezimana P., Rozhmina T.A., Zhuchenko A.A., Povkhova L.V., Pushkova E.N., Novakovskiy R.O., Pavelek M., Vladimirov G.N., Nikolaev E.N. (2020). Genetic diversity of SAD and FAD genes responsible for the fatty acid composition in flax cultivars and lines. BMC Plant Biol..

[67] Miart F., Fontaine J.X., Mongelard G., Wattier C., Lequart M., Bouton S., Molinie R., Dubrulle N., Fournet F., Demailly H. (2021). Integument-Specific Transcriptional Regulation in the Mid-Stage of Flax Seed Development Influences the Release of Mucilage and the Seed Oil Content. Cells.

[68] Lin Y., Ma J., Wu N., Qi F., Peng Z., Nie D., Yao R., Qi X., Slaski J., Yang F. (2022). Transcriptome Study of Rice Roots Status under High Alkaline Stress at Seedling Stage. Agronomy.

[69] Wang N., Lin Y., Qi F., Xiaoyang C., Peng Z., Yu Y., Liu Y., Zhang J., Qi X., Deyholos M. (2022). Comprehensive Analysis of Differentially Expressed Genes and Epigenetic Modification-Related Expression Variation Induced by Saline Stress at Seedling Stage in Fiber and Oil Flax, *Linum usitatissimum* L.. Plants.

[70] Wang N., Zhang D., Wang Z., Xun H., Ma J., Wang H., Huang W., Liu Y., Lin X., Li N. (2014). Mutation of the RDR1 gene caused genome-wide changes in gene expression, regional variation in small RNA clusters and localized alteration in DNA methylation in rice. BMC Plant Biol..

[71] Chang W., Niu Y., Yu M., Li T., Li J., Lu K., Basu C. (2022). qPrimerDB: A Powerful and User-Friendly Database for qPCR Primer Design. PCR Primer Design.

